# The Incidence Rate, High-Risk Factors, and Short- and Long-Term Adverse Outcomes of Fetal Growth Restriction: A Report From Mainland China

**DOI:** 10.1097/MD.0000000000000210

**Published:** 2014-12-12

**Authors:** Jing Liu, Xiao-Feng Wang, Yan Wang, Hua-Wei Wang, Ying Liu

**Affiliations:** From the Department of Neonatology and NICU, Bayi Children's Hospital Affiliated to Beijing Military General Hospital, Beijing 100700, China (JL, X-FW, YW, H-WW, YL); Graduate School of Anhui Medical University, Hefei 230033, China (X-FW, H-WW, YL); Graduate School of Southern Medical University, Guangzhou 510515, China (YW).

## Abstract

To investigate the incidence and high-risk factors of fetal growth restriction **(**FGR) in Mainland China and determine the adverse effects of this condition on fetal-neonatal health.

This study was a retrospective chart review. We investigated the incidence rate of FGR using a retrospective analysis of clinical data obtained from mothers and newborns from 7 hospitals in Mainland China from January 1 to December 31, 2011. The short-term outcomes of FGR were analyzed based on data obtained from the neonatal intensive-care unit (NICU) of Bayi Children's Hospital. The long-term outcomes of FGR were determined after a follow-up study of 125 cases of FGR in children at 18 months. The physical development index, mental development index (MDI), and psychomotor development index (PDI) were compared between FGR patients and controls.

The incidence of FGR was 8.77%. The incidence of FGR was significantly higher in females than in males (9.80% vs 7.84%, *P* < 0.05). The incidence of FGR in preterm infants was higher than that in full-term infants (16.43% vs 7.87%, *P* *<* 0.01). Chronic hypertension, abnormal amniotic fluid, and umbilical cord abnormalities were independent factors of FGR. A significantly higher incidence of complications, including hypoglycemia, asphyxia, hypoxic-ischemic encephalopathy, gastrointestinal bleeding, congenital malformations, polycythemia, lung hemorrhage, apnea, congenital heart disease, and disseminated intravascular coagulation, was observed in FGR patients than in controls. The FGR prolonged the duration of the hospital stay and markedly increased hospitalization expenses (*P* < 0.05). Children with FGR showed catch-up growth, which reached the level of the control group after 1.5 years, but these individuals still had lower MDI and PDI scores.

The incidence rate of FGR in Mainland China was 8.77%. It has a significantly adverse effect on fetal-neonatal health and cognitive development.

## INTRODUCTION

Fetal growth restriction (FGR) is affected by various pathological factors (including the mother, placenta, and fetus) that can lead to restricted growth potential of afflicted individuals. As the mediator of all communications between the mother and the fetus, the placenta plays a key role in the pathways of fetal growth and development. Therefore, placental insufficiency caused by all kinds of reasons is the main mechanism.^1–4^ Placental insufficiency adversely affects total nutrient, blood and oxygen active transfer and reduces glucose, lipids and protein synthesis, which leads to fetal undergrowth.^1–5^ Upon delivery, individuals with FGR are typically considered small for gestational age (SGA) infants (ie, birth weight (BW) is often less than the 10th percentile of the average weight of infants at the same gestational age (GA)).^6^ FGR is associated with increased morbidity and mortality.^6,7^ It adversely affects the nervous system, resulting in long-term neurological sequelae, such as cerebral palsy, exercise and behavioral disorders, a reduced ability to learn and a short attention span.^8–10^ In adulthood, individuals with FGR have a significantly increased risk of type II diabetes, obesity, hypertension, coronary heart disease, dyslipidemia, insulin resistance syndrome (or metabolic syndrome), and other diseases.^11,12^ Therefore, research about FGR is a worldwide issue.^13–16^ In this study, we investigated the incidence rate of FGR and analyzed the effects of this condition on fetal and neonatal near-term health in China. We aim to use the results from this study to develop a working protocol for improving fetal and neonatal health after the occurrence of FGR.

## PATIENTS AND METHODS

The institutional review board of the General Hospital Beijing Military approved the study protocol. Retrospective analysis, multivariate logistic regression analysis and paired design were used to analyze the difference between 2 groups. This investigation includes 3 parts: Part 1: Incidence rate of FGR in Mainland China, Part 2: Short-term outcomes of newborns with FGR, and Part 3: Long-term outcomes of newborns with FGR.

### Part 1: The FGR Incidence Rate in Mainland China

The data were acquired from 7 different hospitals, including Beijing Military General Hospital, Beijing Obstetrics and Gynecology Hospital, Maternal and Child Health Care Center of Shaanxi Province, The First Affiliated Hospital of Xinxiang Medical College, Linyi People's Hospital of Shandong Province, The Affiliated He-Xian Memorial Hospital of Southern Medical University, and The First Affiliated Hospital of Anhui Medical University. These hospitals are located in 4 provinces and 1 municipality in Mainland China. The total number of deliveries and newborns with FGR at all of the hospitals was recorded between January and December 2011. Other information, including the age of the mother at pregnancy, and complications of pregnancy (eg, hypertensive diseases of pregnancy, gestational glucose intolerance, and diabetes), as well as the GA, gender, and BW of the newborns, with or without birth asphyxia or other complications, was also obtained.

### Part 2: Short-Term Outcomes of Newborns With FGR

The short-term outcomes of this study deal with the outcomes within 28 days after birth (ie, within the neonatal period). Neonates admitted to the Department of Neonatology and Neonatal Intensive Care Unit at Bayi Children's Hospital Affiliated with Beijing Military General Hospital between January and December 2011 were considered for inclusion in the present study. As the largest neonatal intensive care unit (NICU) in China (with 350 beds), more than half of the patients from Mainland China are admitted to this NICU. Therefore, the data from this NICU have good generalizability. Characteristics of the patients, including GA, gender, BW, body length (BL), head circumference (HC), the major cause of hospitalization, the duration of hospitalization, in-hospital costs, prognosis, and maternal health conditions, were recorded for each newborn.

### Part 3: Long-Term Outcomes of Newborns With FGR: Follow-Up at 18 Months of Life

A total of 125 infants born with FGR were investigated at 18 months of life, and an additional 125 newborns who were considered appropriate for gestational age (AGA) served as controls. Physical development, the mental development index (MDI) and the psychomotor development index (PDI) were compared between the 2 groups. No differences in general information, including GA at birth, Apgar scores at 1 and 5 minutes of birth, maternal characteristics, delivery mode, family socioeconomic status, and parents’ educational background, were detected between these 2 groups (*P* > 0.05).

#### Physical Development

Included BL (accurate to 0.1 cm), HC (accurate to 0.1 cm), and BW (accurate to 0.1 kg) were measured in every child.

#### MDI and PDI Testing

The Bayley Scales of Infant Development, Second Edition (BSID-II), were used to assess every child, producing MDI and PDI scores.^17^ The MDI evaluates memory, habituation, problem solving, and language. The PDI evaluates the control of gross muscle groups, including movements associated with standing, walking, running, and jumping. The PDI also assesses fine motor manipulations involved in prehension, the adaptive use of writing implements, and imitation of hand movements. An assessor scored the items in both tests based on the elicited and observed behavior, and not according to parental reports.

### Statistical Analysis

Data analysis was conducted using SPSS for Windows (Release 16.0, SPSS, Chicago, IL). All data with a normal distribution are presented as the mean ± standard deviation (*x* ± s). The *t*test was used for comparison between the 2 groups. Non-normally distributed data are expressed as medians, and the Kruskal–Wallis test was used for comparison between the 2 groups. The χ^2^ test was used for comparison of enumeration data. Multivariate logistics regression analysis was used to evaluate the risk factors associated with FGR and calculate the odd ratios (ORs) and 95% confidence intervals (CIs). *P* < 0.05 was considered statistically significant. The information reported been evaluated by an expert.

## RESULTS

### The FGR Incidence Rate in Mainland China

A total of 3106 newborns with FGR were identified among the 35,418 deliveries at the 7 hospitals during the study period. The incidence of FGR in this population was 8.77%. The incidence of FGR was significantly higher in preterm infants (with GA < 37 weeks) than in full-term infants (with GA ≥37 weeks) (16.43% vs 7.87%, χ^2^ = 235.5, *P* < 0.01). The incidence of FGR was significantly higher in females (12.75%, 358/2805) than in males (7.75%, 321/4140) (χ^2^ = 76.37, *P* < 0.05) (Table 1).

**TABLE 1:**
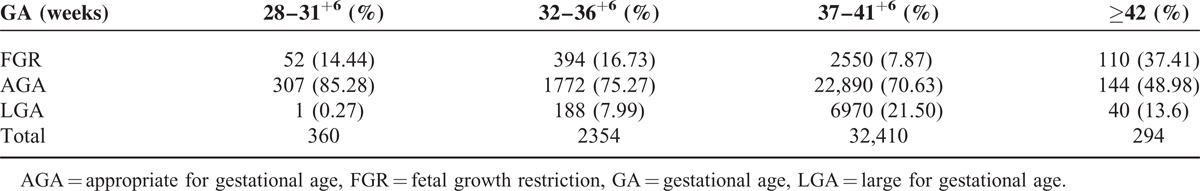
The Incidence of FGA at Different Gestational Ages

### High-Risk Factors of FGR and Multivariate Logistic Regression Analysis

Analysis of risk factors for FGR showed that the incidence of FGR in females was significantly higher than that in males (9.80% vs 7.84%, χ^2^ = 6.285, *P* < 0.05). Notably, the incidence of FGR was significantly higher in pregnant women suffering from pregnancy-induced hypertension, abnormal amniotic fluid volume, and umbilical cord abnormalities than in those without these complications (χ^2^ = 4.850–36.771, *P* < 0.05). The incidence of FGR in women who had anemia during pregnancy and other aspects of cardiovascular disease was slightly higher compared with that in the IUGR group, but this difference was not significant (Table 2).

**TABLE 2:**
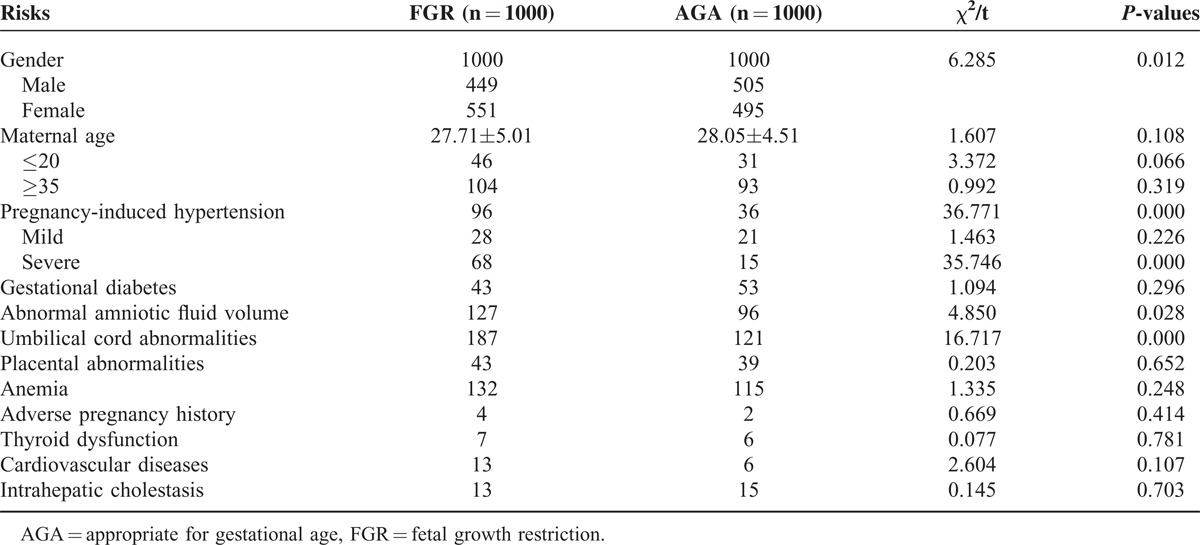
The Single Factor Analysis for High Risks of FGR

To further investigate the relationship between various risk factors and FGR, we used multiple regression and stepwise discriminant analyzes to determine whether FGR was a dependent variable, defined as YES = 1 and NO = 2. Univariate analysis showed 5 independent risk factors that were associated with FGR. Logistic regression analysis showed that pre-eclampsia, umbilical cord abnormalities, and abnormal amniotic fluid volume were high-risk factors independently associated with FGR (Table 3).

**TABLE 3:**
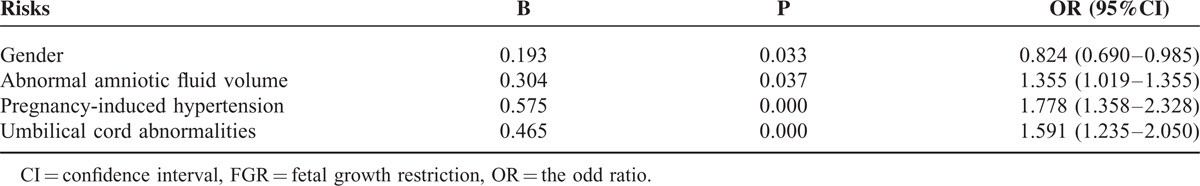
The Multivariate Logistic Regression Analysis for FGR

### Short-Term Adverse Outcomes of Newborns With FGR

A total of 6948 patients were treated at the Department of Neonatology and NICU from January to December 2011. Of these, 679 patients (9.77%) suffered from FGR, with an average GA of 36.3 ± 3.03 weeks, and the mean BW was 1972.22 ± 502.11 g.

After birth, newborns with FGR often develop considerable problems in multiple systems, and the neonatal mortality rate is much greater than that for weight-matched neonates. In the present study, comparison with AGA infants showed the following observations: FGR newborns had a higher morbidity, associated with hypoglycemia, birth asphyxia, and brain injury, including hypoxic-ischemic encephalopathy, intracranial hemorrhage, periventricular leukomalacia, gastrointestinal bleeding, congenital malformations, polycythemia, pulmonary hemorrhage, apnea, disseminated intravascular coagulation, and hyperbilirubinemia. There were no significant differences in pneumonia, respiratory distress syndrome, sepsis, intracranial infection, necrotizing enterocolitis, and meconium aspiration syndrome between the groups (Table 4). The duration of hospitalization for FGR newborns was longer (16, 11–24 days) than that for AGA infants (13, 10–17 days; Z = 7.07, *P* < 0.01). Therefore, for FGR newborns, hospitalization was prolonged by 23.1% compared with AGA infants. The mortality rates among FGR and AGA infants were 0.74% (5/679) and 0.1% (1/1000), respectively (χ^2^ = 11.37, *P* < 0.01). Therefore, the mortality rate in FGR newborns was 7.4 times that of those with AGA. The average in-hospital cost was significantly higher (16,000, 11,400–25,500 Yuan RMB) for FGR patients than for AGA infants (12,700, 9375–18,300 Yuan RMB), representing a 26% increase (Z = 7.89, *P* < 0.01).

**TABLE 4:**
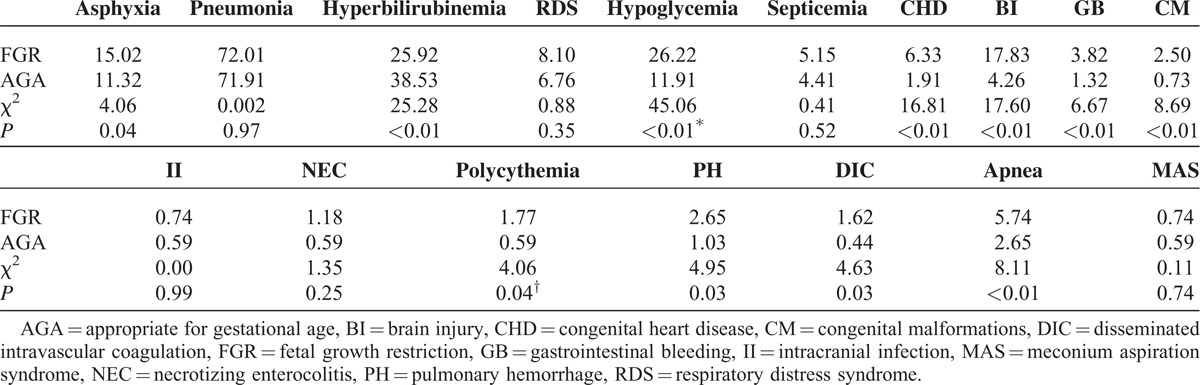
Influence of FGR on Fetal-Neonatal Health (%)

### Long-Term Adverse Outcomes for Newborns With FGR

Physical development of FGR patients was delayed but eventually reached that of children in the control group after 1.5 years (Table 5). However, the MDI and PDI scores showed that FGR children remained underdeveloped. Indeed, after 1.5 years, the number of patients with MDI scores less than 70 in the FGR and AGA groups was 8 (6.4%) and 1 (0.8%) (χ^2^ = 0.429, *P* *=* 0.531), respectively; the number of patients with PDI scores less than 70 in the FGR and AGA groups was 12 (9.6%) and 2 (1.6%) (χ^2^ = 0.244, *P* *=* 0.621), respectively.

**TABLE 5:**
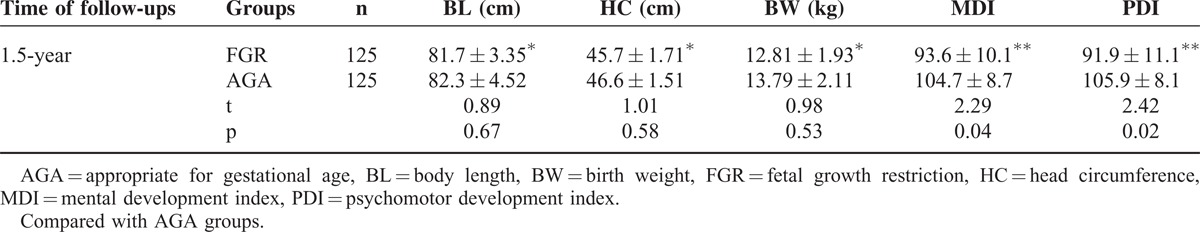
The Influence of FGR on Child Physical and Cognitive Development at 1.5-Year of Age *X*¯ ± *S*

## DISCUSSION

The present study showed that the incidence rate of FGR in Mainland China is 8.77%, which is similar to the developed world.^7,18–20^ The incidence of FGR in preterm infants was significantly higher than that in full-term neonates. Approximately, 20,000,000 pregnancies per year are reported in China, and FGR complicates nearly 1,600,000 births annually. Therefore, improving antenatal health care to reduce the incidence of FGR is important because FGR can lead to fetal death, stillbirth, and other fetal and neonatal diseases.

The current study showed that GA, gender, pregnancy-induced hypertension/pre-eclampsia, umbilical cord abnormalities, and abnormal amniotic fluid volume were high-risk factors independently associated with FGR. We observed that the incidence of FGR was significantly higher in females than in males. This finding is consistent with that by Graner et al.^21^, who reported that females have a higher incidence of FGR than males (OR = 1.61, 95% CI: 1.27–2.03). Pregnancy-induced hypertension/pre-eclampsia causes systemic small artery spasm perfusion abnormalities, resulting in in utero insufficiency, a reduction in placental blood flow, placental dysfunction, affecting fetal oxygenation, nutrient absorption, and consequently fetal development, resulting in FGR. Oligohydramnios and meconium-stained amniotic fluid are associated with placental insufficiency. Placental insufficiency might lead to poor blood circulation in the placenta and nutrient deficiency, thereby affecting the growth of the fetus and leading to FGR. Many preterm infants also exhibit FGR, with an increased risk for perinatal death and neonatal complications.^7^ Therefore, improving maternal and perinatal health care is important. The high-risk factors for FGR are quite different from those in developed countries where maternal smoking is one of the most important contributors to FGR^22^. This may be because Chinese woman have lower rates of smoking.

The present study also showed that FGR might adversely affect fetal-neonatal near-term health, including:

1. increased disease morbidity, associated with hypoglycemia (the most common metabolic disorder^23^), birth asphyxia, brain injury, hyperbilirubinemia, polycythemia, pulmonary hemorrhage, apnea, and disseminated intravascular coagulation (these diseases contribute to increased perinatal mortality);
2. increased infant mortality from 0.1% in AGA infants to 0.74% in FGR infants (a 7.4-fold increase in this group);
3. prolonged hospitalization (increased 23.1% in this study); and
4. increased in-hospital costs (increased 22.6% in this study).

Recently, long-term neurological sequelae have received much attention from clinicians.^8,9^ Our study showed that acute brain injury, such as hypoxic-ischemic encephalopathy, periventricular leukomalacia, and intracranial hemorrhage, is also significantly increased in FGR newborns. Therefore, clinicians need to monitor brain damage in FGR newborns in the future.

Catch-up growth is typically observed in FGR newborns after birth. Our follow-up results showed that the physical development of FGR children reached the level of that in the control group after 1.5 years. Indeed, there were no significant differences in BL, HC, and body weight between the FGR and AGA groups. This finding suggests that it takes 1.5 to 2 years to improve the growth of newborns with FGR. Nevertheless, the MDI and PDI scores for FGR children remained significantly lower than those for AGA children; the incidence of MDI and PDI scores lower than 70 in the FGR group was 8 and 6 times higher, respectively, than that in the AGA group. Therefore, monitoring cognitive development of these infants for longer periods is important. Indeed, catch-up growth typically results in increased abdominal adipose tissue with less-than-normal development of lean body mass, associated with adult morbidities, such as obesity and type II diabetes, and reproductive endocrine abnormalities.^21^ These issues are important for front-line workers and health-care planners.

## CONCLUSIONS

In conclusion, FGR is a common condition observed in Mainland China. The general incidence of FGR is 8.77%. It is significantly higher in preterm infants (16.43%) than in full-term infants (7.87%). It is higher in females (12.75%) than in males (7.75%). Newborns with FGR have higher morbidity and mortality, with longer hospital stays and higher hospital costs than AGA infants. Although the physical development of FGR infants can reach the level of controls after 1.5 years, these children typically show under-developed cognitive function. These results provide valuable information for medical-health care practitioners. However, this study has also some limitations. First, the data does not include those geographically remote areas. This may lead to some bias. Second, the study does not examine when the neuropsychological development of FGR children can catch up to normal levels. Thus, further investigations are needed to assess the above conditions.
